# Severity of acne vulgaris predicts maladaptive psychosocial perceptions and neuropsychiatric disorders: a case-control study

**DOI:** 10.1007/s00403-025-04123-z

**Published:** 2025-03-19

**Authors:** Brad R. Woodie, Isaiah N. Holt, Justin A. Freking, Heather C. W. Harrison, Gabrielle M. Rivin, Alan B. Fleischer Jr

**Affiliations:** 1 College of Medicine, University of Cincinnati College of Medicine, Cincinnati, OH USA; 2 School of Professional Psychology, Spalding University, Louisville, KY USA; 3 Department of Dermatology, University of Cincinnati College of Medicine, Cincinnati, OH USA

**Keywords:** Social determinants of health, Patient-reported outcome measures, Psychodermatology, Social stigma, Self concept

## Abstract

Acne vulgaris can negatively impact a patient’s quality of life. This study evaluated the psychological and interpersonal perceptions of patients who had received acne treatment. Using data from the National Institutes of Health’s *All of Us* Research Program, we analyzed survey responses from 1,843 participants treated with systemic therapies for acne (reflecting more severe disease) and 1,843 matched participants treated with topical therapies alone. Outcomes included surveys assessing both mental and social health and the prevalence of neuropsychiatric conditions between groups. Compared with those who used only topical therapies, participants treated with systemic therapies for acne experience greater personal and relational difficulties in addition to having higher rates of obsessive-compulsive disorder, personality disorders, anxiety, and depression. These findings were robust in a sensitivity analysis excluding isotretinoin-treated participants. The subset of people with acne who receive systemic therapy comprise a high-risk group for adverse psychological outcomes. We aim to bring greater awareness and sensitivity to these issues among dermatologists and other clinicians managing acne. We also aim to highlight the potential need for neuropsychiatric support in patients with more severe acne.

**Supplementary Information:**

The online version contains supplementary material available at 10.1007/s00403-025-04123-z.

## Introduction

The impact of acne vulgaris extends beyond physical appearance; it is associated with perceptions of unattractiveness, sadness, loneliness, and unhealthiness [10]. Acne has been linked to psychosocial stress, diagnosed mental disorders, and even suicidal ideation [7, 13, 15, 16]. Stress, in turn, is linked with worse disease severity, creating a potential feedback loop [11].

Though onset is typically in adolescence, acne frequently persists into adulthood and may be considered a chronic condition [2, 4, 20]. Acne is nearly universal in adolescence [2], but Corey et al. found that few affected individuals seek medical care [3]. This low rate of care-seeking behavior introduces the potential for confounding in understanding the psychological burden of acne.

Extant research on the psychodermatology of acne largely compares individuals with acne to unaffected controls. Therefore, we aimed to evaluate the mental and interpersonal impairment that acne has on the lives of patients with the disease while accounting for the potential confounding in care utilization. Using validated surveys in a diverse sample of individuals, we evaluated the psychological and interpersonal burden of acne specifically within patients who had previously received a formal diagnosis of the condition. By stratifying by treatment strength, we intended to explore the connection between disease severity and mental health in acne patients. These results may help guide clinicians’ decisions surrounding psychological screening while initiating or escalating acne therapy.

## Methods

We analyzed de-identified participant data from the National Institutes of Health’s *All of Us* Research Program version 7, which includes data from 2017 to 2022. We identified 4,790 participants with an acne diagnosis (Systematized Nomenclature of Medicine Clinical Terms SNOMED code 11381005) in their electronic medical record who also completed the “Overall Health” and “Social Determinants of Health” survey questions. This study followed the Strengthening the Reporting of Observational Studies in Epidemiology (STROBE) reporting guidelines.

Guidelines recommend topical medications for mild acne and additional systemic antibiotics, spironolactone, or isotretinoin for moderate-to-severe acne [12, 14]. We hypothesized that patients treated with systemic therapy have a higher visible and/or psychosocial disease burden compared to patients treated with only topical medications and can therefore be considered as having “more severe” acne.

A propensity-matched case-control design was used. Systemic therapy for acne (a “case”) was defined as receiving one of the following drugs at a visit for acne: oral doxycycline, minocycline, sarecycline, azithromycin, erythromycin, spironolactone, or isotretinoin. Although several are approved in the United States for treating acne, we did not consider oral contraceptives because we were unable to determine if they were primarily prescribed for acne. The controls in this study were participants also with an acne diagnosis but who received topical therapies only. Controls were 1:1 propensity matched to cases on age, sex, race, ethnicity, and income for a final sample of 1,843 participants who received systemic therapy for acne (38% of all patients with an acne diagnosis) and 1,843 participants who used only topical therapy.

To compare the psychosocial status of people based on their acne therapy categorization, we selected several surveys collected by *All of Us*. The *All of Us* program uses the Patient-Reported Outcomes Measurement Information System (PROMIS) Global Health survey to assess overall mental, social, and physical health [17]. We selected the overall mental and social health questions. Questions and scoring of responses are shown in Online Resource 1. To evaluate social determinants of health, the *All of Us* program includes several instruments that have been previously validated [19]. Of these, we selected the University of California Los Angeles Loneliness Scale, Cohen’s Perceived Stress Scale, the Everyday Discrimination scale, and the Everyday Discrimination in Medical Settings scale. Scoring of these scales is explained in detail by Tesfaye et al. [19]. We also compared the frequency of smoking, vaping, and alcohol use which are recorded as “not at all,” “some days,” and “every day.”

Additionally, we evaluated the presence of several neuropsychiatric conditions between groups using Systematized Nomenclature of Medicine (SNOMED) codes. SNOMED is a standardized clinical terminology system used to hierarchically organize information across electronic medical records. Conditions of interest included anxiety disorder (SNOMED 197480006), major depressive disorder (370143000), post-traumatic stress disorder (PTSD) (47505003), insomnia (193462001), obsessive-compulsive disorder (OCD) (191736004), attention-deficit hyperactivity disorder (ADHD) (406506008), personality disorder (33449004), psychotic disorder (69322001), somatoform disorder (31297008), and bipolar disorder (13746004).

Independent samples T-tests were performed on aggregated scale scores‌ or individual question responses. Univariable logistic regression was performed to compare the likelihood of neuropsychiatric diagnoses. χ^2^ tests were performed on smoking, vaping, and alcohol use. To evaluate the potential impact of patients with the most severe acne on our findings, a sensitivity analysis was performed without the patients who received isotretinoin (*n* = 185). Significance was set at two-tailed *p* <.05. Analysis was performed in the SAS Studio environment within the All of Us Researcher Workbench.

## Results

Characteristics of the propensity-matched cohorts are shown in Table 1. Compared with topically treated patients, participants who received systemic therapy for acne reported more loneliness, perceived stress, perceived discrimination, perceived discrimination in healthcare, and trouble with emotional problems (Table 2). Participants who received systemic therapy for acne also reported a lower quality of life, mental health, social satisfaction, and ability to complete social roles.

**Table 1:** Demographics of participants with acne treated with systemic therapy and propensity score matched participants with acne treated with only topical therapy. Topical and systemic therapies are listed as well but not formally compared

Variable	Topical therapy only (*N* = 1843) (mean and 95% CI or n, %)	Systemic therapy (*N* = 1843) (mean and 95% CI or n, %)	*P*-value
Age (years)	49.6 (48.9, 50.4)	48.8 (48.0, 49.5)	0.1
Income ($)	64,200 (63500, 64900)	64,000 (63400, 64700)	0.7
Sex			1.0
Female	1477 (39.4%)	1519 (40.5%)	
Male	333 (8.9%)	351 (9.4%)	
Other	33 (0.9%)	35 (0.9%)	
Race^a^			0.9
Asian	48 (1.3%)	44 (1.2%)	
Black	161 (4.3%)	164 (4.4%)	
Other	212 (5.7%)	214 (5.7%)	
White	1422 (37.9%)	1483 (39.6%)	
Ethnicity			0.4
Hispanic or Latino	171 (4.6%)	141 (3.8%)	
Not Hispanic or Latino	1613 (43.1%)	1701 (45.4%)	
Other	59 (1.6%)	62 (1.6%)	
Therapy^b^			--
Benzoyl peroxide	560 (30.4%)	719 (39.0%)	
Topical antibiotic^c^	1038 (56.3%)	999 (54.2%)	
Topical retinoid^d^	1124 (61.0%)	940 (51.0%)	
Topical sulfur	138 (7.5%)	358 (19.4%)	
Azelaic acid	53 (2.9%)	55 (3.0%)	
Clascoterone	< 20^g^	< 20	
Oral tetracycline^e^	-	1530 (83.0%)	
Oral macrolide^f^	-	686 (37.2%)	
Spironolactone	-	267 (14.5%)	
Isotretinoin	-	185 (10.0%)	

^a^ Race and ethnicity were obtained from self-report and were included in this analysis as a goal of *All of Us* to collect and report data from a diverse group of participants. “Other” races included American Indian or Alaskan Native, Middle Eastern or North African, Native Hawaiian or Other Pacific Islander, and more than one population

^b^ Percentages sum to more than 100% because all therapies for acne for each participant are represented. Additionally, combination preparations (such as benzoyl peroxide/clindamycin) were counted in all applicable categories

^c^ Topical clindamycin, erythromycin, sulfacetamide, or dapsone

^d^ Adapalene, tretinoin, or trifarotene. Tazarotene was not used in the sampled participants

^e^ Doxycycline, minocycline, or sarecycline

^f^ Azithromycin or erythromycin

^g^ To protect patient privacy, counts less than 20 are hidden

**Table 2:** Measures of personal and social stress between participants with acne prescribed systemic therapy^a^ versus propensity-matched participants with acne who only received topical therapy

Variable	Topical therapy only (*N* = 1843) Mean (95% CI)	Systemic therapy (*N* = 1843) Mean (95% CI)	Mean Difference (95% CI)	T-test *P*-value
Self-rated Mental Health	3.53 (3.49, 3.58)	3.41 (3.37, 3.46)	0.12 (0.06, 0.19)	**0.0003**
Self-rated trouble with Emotional Problems*^b^	2.50 (2.45, 2.55)	2.62 (2.57, 2.67)	-0.12 (-0.19, -0.05)	**0.0005**
Perceived Stress Scale*	30.3 (30.1, 30.5)	30.7 (30.5, 30.9)	-0.42 (-0.69, -0.15)	**0.002**
Self-rated Quality Of life	3.77 (3.73, 3.82)	3.68 (3.64, 3.73)	0.09 (0.03, 0.15)	**0.004**
Perceived Discrimination Scale*	0.88 (0.85, 0.92)	0.96 (0.92, 1.00)	-0.07 (-0.13, -0.02)	**0.008**
Perceived Discrimination in Healthcare Scale*	1.59 (1.56, 1.62)	1.64 (1.62, 1.67)	-0.05 (-0.09, -0.01)	**0.01**
Self-rated Ability to Complete Social Roles	3.87 (3.82, 3.91)	3.79 (3.74, 3.83)	0.08 (0.02, 0.14)	**0.01**
Loneliness Scale*	59.3 (58.8, 59.7)	60.0 (59.5, 60.6)	-0.74 (-1.46, -0.03)	**0.04**
Self-rated Social Satisfaction	3.54 (3.49, 3.59)	3.46 (3.42, 3.51)	0.07 (0.01, 0.14)	**0.04**

^a^ Systemic therapy was identified by a prescription for oral doxycycline, minocycline, sarecycline, azithromycin, erythromycin, spironolactone, or isotretinoin at a visit for acne

^b^ Scales marked with * use higher scores to represent greater severity

Several neuropsychiatric conditions were more likely in those who received systemic therapy for acne (Table 3). OCD showed the strongest association, with personality disorders, anxiety, depression, ADHD, and PTSD having significant associations as well. There was no significant difference in the frequency of smoking, vaping, or alcohol consumption between groups (Table 4).

**Table 3:** Logistic regression for the presence of diagnosed psychiatric conditions between participants who received systemic therapy for acne (oral Doxycycline, Minocycline, Sarecycline, Azithromycin, spironolactone, or isotretinoin) versus propensity-matched participants with acne who only received topical therapy

Condition	*N*, topical therapy	*N*, systemic therapy	Odds Ratio	95% CI	*p*-value
**Anxiety disorder**	755	946	1.42	1.25–1.62	**< 0.0001**
**Depressive disorder**	796	979	1.39	1.22–1.58	**< 0.0001**
**Personality disorder**	68	113	1.65	1.21–2.24	**0.002**
**Post-Traumatic Stress Disorder**	227	303	1.35	1.12–1.62	**0.002**
**Obsessive-Compulsive Disorder**	33	60	1.78	1.16–2.74	**0.008**
**Attention-Deficit Hyperactivity Disorder**	141	193	1.36	1.08–1.71	**0.008**
Eating disorder	57	80	1.37	0.97–1.94	0.07
Bipolar disorder	92	121	1.29	0.98–1.71	0.07
Psychotic disorder	63	86	1.34	0.96–1.86	0.09
Somatoform disorder	65	83	1.25	0.90–1.74	0.2
Insomnia	438	482	1.09	0.94–1.26	0.3

**Table 4:** No significant difference in the frequency of smoking, vaping, or drinking alcohol

Variable	Level	Topical therapy only (*n*, %)	Systemic therapy (*n*, %)	Total (*n*, %)	Χ^2^*p*-value
Smoking Frequency	Not at all	1739 (46.4%)	1773 (47.3%)	3512 (93.7%)	0.2
	Some days	38 (1.0%)	41 (1.1%)	79 (2.1%)	
	Every day	66 (1.8%)	91 (2.4%)	157 (4.2%)	
Vaping Frequency	Not at all	251 (39.4%)	253 (39.7%)	504 (79.1%)	0.1
	Some days	34 (5.3%)	42 (6.6%)	76 (11.9%)	
	Every day	19 (3.0%)	38 (6.0%)	57 (9.0%)	
Alcohol Frequency	None in past year	236 (6.6%)	226 (6.3%)	462 (12.9%)	0.4
	Once per month	554 (15.5%)	610 (17.1%)	1164 (32.6%)	
	2 to 4 per month	438 (12.3%)	449 (12.6%)	887 (24.8%)	
	2 to 3 per week	314 (8.8%)	342 (9.6%)	656 (18.4%)	
	4 + per week	214 (6.0%)	192 (5.4%)	406 (11.4%)	

The sensitivity analysis was consistent with the primary results, indicating that the exclusion of isotretinoin-treated patients did not substantially alter our findings. (Online Resources 2 and 3). Social satisfaction was no longer significantly different (difference of means 0.07 [95% confidence interval − 0.002, 0.14], *p* =.06) and bipolar disorder was newly associated (odds ratio 1.4 [95% CI 1.05–1.85], *p* =.02).

## Discussion

We found that people who previously received systemic treatment for acne (which we considered as more severe acne) experience more internal and interpersonal stress compared to those who received topical treatments only. Additionally, people who received systemic treatment for acne are more likely to be diagnosed with certain neuropsychiatric disorders.

This study evaluates the small subset of people with acne who received systemic therapy and finds that they experience more psychosocial stress relative to those with acne treated only topically. Corey et al. found that only 17% of people with acne sought medical care for it [3], and the current study found that only 38% of people with formal acne diagnoses received systemic treatment. Despite this, acne is one of the most common skin conditions managed by dermatologists and non-dermatologists alike [5]. Due to the cumulative burden experienced across multiple domains of mental health, we suggest that clinicians involved in managing patients with acne maintain a high index of suspicion for psychosocial difficulties when escalating to systemic therapy. People with acne often try several topical medications prior to systemic therapy and can become frustrated with a perceived “trial and error” process [8]. This could contribute to feelings of loneliness and hopelessness. As we found that this group also perceives higher levels of discrimination in healthcare settings, taking a patient-centered and nonjudgmental approach to care may be especially important. This study cannot determine causality, but it is possible that psychological comorbidities influence patient preference for systemic treatments. It also remains unclear if patients having certain pre-existing psychological comorbidities are more likely to desire systemic treatment independent of their visually apparent acne severity. A feedback loop is also possible, in which acne and a mental health disorder are mutually exacerbated. Regardless, patients who receive systemic treatment have higher ratings of psychosocial impairment like loneliness and difficulty in relationships.

Acne is well-known to be associated with internal and interpersonal stress [13, 15, 16], but comparisons considering the role of acne severity have been limited. Prior work has identified severity-dependent odds of depression, social impairment, and suicidal ideation [6, 18]. Our work adds to these findings by using a larger and more diverse sample and using a wider array of outcome measures. Previous studies have demonstrated associations between acne and increased prevalence of OCD, depression, anxiety, and ADHD [1, 9, 15]. Our data expands on these relationships by considering the severity of acne and additionally identifies personality disorders and PTSD as being associated with more severe acne. Systemically treated acne had the strongest association with OCD, and prior work has demonstrated increases in obsessions and compulsions related to the unpredictable nature of acne [1]. The newly identified and previously established relationships between neuropsychiatric comorbidities and systemically treated acne underscore the value of considering such conditions in patients with acne treated systemically.

This study has limitations. Although the *All of Us* participant base is diverse in race, ethnicity, sexuality, and gender, it is not a nationally representative sample. We also cannot account for the temporal relationship between when systemic therapy was initiated, completed, and when the survey responses were recorded. Additionally, we were unable to identify a known minimal clinically important difference for the surveys in this study. We also could not assess the duration of treatment or long-term physical complications such as cosmetically sensitive scarring. A potential limitation of the study is that we did not have a visual severity assessment for participants. However, even when assessed, objective standards for acne severity may not reflect a patient’s subjective experience. Future research may address these limitations through the use of a longitudinal design and subjective as well as objective measures of acne severity.

By leveraging a large and diverse dataset, we demonstrated that individuals receiving systemic treatment for acne face greater internal and interpersonal challenges and are more likely to have certain neuropsychiatric conditions. These findings emphasize the importance of a holistic approach to acne management, where clinicians proactively screen for psychosocial and mental health concerns alongside dermatologic treatment. This approach can help optimize patient care by ensuring timely psychological support for those most at risk, ultimately improving both dermatologic and mental health outcomes.

## Electronic supplementary material

Below is the link to the electronic supplementary material.

Supplementary Material 1

## Data Availability

This study used the All of Us Research Program’s Controlled Tier Dataset version 7, available to authorized users on the Researcher Workbench.
