# Supplementary material for: Severity of acne vulgaris predicts maladaptive psychosocial perceptions and neuropsychiatric disorders: a case-control study
Source: Arch Dermatol Res. 2025 Mar 19;317(1):597. doi: 10.1007/s00403-025-04123-z (PMC11922974; doi:10.1007/s00403-025-04123-z)
Supplement: Supplementary file 1 — Supplementary Material 1 [file 403_2025_4123_MOESM1_ESM.docx]

**Online Resources**

**Online Resource 1: Individual survey questions accessed in the study.**

| **Description** | **Full question** | **Responses*** |
| --- | --- | --- |
| Self-rated Mental Health | In general, how would you rate your mental health, including your mood and your ability to think? | Poor, fair, good, very good, excellent |
| Self-rated Social Satisfaction | In general, how would you rate your satisfaction with your social activities and relationships? | Poor, fair, good, very good, excellent |
| Self-rated Ability to Complete Social Roles | In general, please rate how well you carry out your usual social roles. (This includes activities at home, at work and in your community, and responsibilities as a parent, child, spouse, employee, friend, etc.) | Poor, fair, good, very good, excellent |
| Self-rated Quality Of life | In general, would you say your quality of life is… | Poor, fair, good, very good, excellent |
| Self-rated trouble with Emotional Problems | In the past 7 days, how often have you been bothered by emotional problems such as feeling anxious, depressed or irritable? | Never, rarely, sometimes, often, always |

*For each question, the five-level ordinal responses were transformed into a numeric 1-5.

**Online Resource 2: Measures of mental and social stress between participants with acne prescribed systemic therapy (excluding isotretinoin) versus propensity-matched participants with acne who only received topical therapy.**

| **Variable** | **Topical therapy only (N=1843) Mean (95% CI)** | **Systemic therapy (N=1843) Mean (95% CI)** | **Mean Difference (95% CI)** | **T-test P-value** |
| --- | --- | --- | --- | --- |
| **Self-rated Mental Health** | 3.53 (3.49, 3.58) | 3.42 (3.37, 3.47) | 0.11 (0.05, 0.18) | **.001** |
| **Perceived Stress** | 30.3 (30.1, 30.5) | 30.7 (30.5, 30.9) | -0.43 (-0.70, -0.15) | **.002** |
| **Self-rated quality of life** | 3.77 (3.73, 3.81) | 3.67 (3.63, 3.72) | 0.10 (0.04, 0.16) | **.002** |
| **Self-rated trouble with Emotional Problems** | 2.50 (2.46, 2.55) | 2.61 (2.56, 2.66) | -0.11 (-0.18, -0.04) | **.003** |
| **Perceived Discrimination** | 0.89 (0.85, 0.92) | 0.96 (0.92, 1.00) | -0.07 (-0.13, -0.02) | **.01** |
| **Perceived Discrimination in Healthcare** | 1.59 (1.56, 1.62) | 1.65 (1.62, 1.68) | -0.05 (-0.09, -0.01) | **.01** |
| **Ability to complete Social Roles** | 3.86 (3.82, 3.90) | 3.77 (3.73, 3.82) | 0.09 (0.02, 0.15) | **.01** |
| **Loneliness** | 59.3 (58.8, 59.7) | 60.0 (59.5, 60.6) | -0.72 (-1.45, -0.01) | **.04** |
| Social Satisfaction | 3.54 (3.49, 3.59) | 3.47 (3.42, 3.52) | 0.07 (-0.002, 0.14) | .06 |

*Systemic therapy was identified by a prescription for oral doxycycline, minocycline, sarecycline, azithromycin, erythromycin, or spironolactone at a visit for acne.

**Online Resource 3: Logistic regression for the presence of diagnosed psychiatric conditions between participants who received systemic therapy (excluding isotretinoin) for acne versus propensity-matched participants with acne who only received topical therapy.**

| **Response Variable** | **Odds Ratio** | **95% Confidence Interval** | **p-value** |
| --- | --- | --- | --- |
| **Anxiety disorder (n=1614)** | 1.41 | 1.23 - 1.60 | **<.0001** |
| **Depressive disorder (n=1696)** | 1.42 | 1.25 - 1.62 | **<.0001** |
| **Personality disorder (n=174)** | 1.76 | 1.29 - 2.41 | **.0004** |
| **Post-Traumatic Stress Disorder (n=509)** | 1.41 | 1.17 - 1.70 | **.0004** |
| **Obsessive-Compulsive Disorder (n=88)** | 1.77 | 1.14 - 2.74 | **.01** |
| **Bipolar disorder (n=208)** | 1.40 | 1.05 - 1.85 | **.02** |
| **Attention-Deficit Hyperactivity Disorder (n=311)** | 1.29 | 1.02 - 1.63 | **.03** |
| Psychotic disorder (n=144) | 1.40 | 1.00 - 1.96 | .05 |
| Eating disorder (n=126) | 1.32 | 0.92 - 1.89 | .1 |
| Somatoform disorder (n=143) | 1.31 | 0.93 - 1.83 | .1 |
| Insomnia (n=885) | 1.12 | 0.97 - 1.31 | .1 |

*Systemic therapy was identified by a prescription for oral doxycycline, minocycline, sarecycline, azithromycin, erythromycin, or spironolactone at a visit for acne.
